# In vitro effects of alendronate on fibroblasts of the human rotator cuff tendon

**DOI:** 10.1186/s12891-019-3014-1

**Published:** 2020-01-11

**Authors:** Chang-Meen Sung, Ra Jeong Kim, Young-Sool Hah, Ji-Yong Gwark, Hyung Bin Park

**Affiliations:** 1 Department of Orthopaedic Surgery, Gyeongsang National University College of Medicine, Jinju, South Korea; 2 Department of Convergence Medical Science, Gyeongsang National University, Jinju, South Korea; 3 Institute of Health Sciences, Gyeongsang National University School of Medicine and Biomedical Research Institute, Gyeongsang National University Hospital, Jinju, South Korea; 4 Department of Orthopaedic Surgery, Gyeongsang National University School of Medicine and Gyeongsang National University Changwon Hospital, Changwon, Republic of Korea 51472

**Keywords:** Human rotator cuff tendon fibroblasts, Alendronate, Wound healing

## Abstract

**Background:**

Bone mineral density of the humeral head is an independent determining factor for postoperative rotator cuff tendon healing. Bisphosphonates, which are commonly used to treat osteoporosis, have raised concerns regarding their relationships to osteonecrosis of the jaw and to atypical fracture of the femur. In view of the prevalence of rotator cuff tear in osteoporotic elderly people, it is important to determine whether bisphosphonates affect rotator cuff tendon healing. However, no studies have investigated bisphosphonates’ cytotoxicity to human rotator cuff tendon fibroblasts (HRFs) or bisphosphonates’ effects on rotator cuff tendon healing. The purpose of this study was to evaluate the cytotoxicity of alendronate (Ald), a bisphosphonate, and its effects on HRF wound healing.

**Methods:**

HRFs were obtained from human supraspinatus tendons, using primary cell cultures. The experimental groups were control, 0.1 μM Ald, 1 μM Ald, 10 μM Ald, and 100 μM Ald. Alendronate exposure was for 48 h, except during a cell viability analysis with durations from 1 day to 6 days. The experimental groups were evaluated for cell viability, cell cycle and cell proliferation, type of cell death, caspase activity, and wound-healing ability.

**Results:**

The following findings regarding the 100 μM Ald group contrasted with those for all the other experimental groups: a significantly lower rate of live cells (*p* < 0.01), a higher rate of subG1 population, a lower rate of Ki-67 positive cells, higher rates of apoptosis and necrosis, a higher number of cells with DNA fragmentation, higher caspase-3/7 activity (*p* < 0.001), and a higher number of caspase-3 positive staining cells. In scratch-wound healing analyses of all the experimental groups, all the wounds healed within 48 h, except in the 100 μM Ald group (*p* < 0.001).

**Conclusions:**

Low concentrations of alendronate appear to have little effect on HRF viability, proliferation, migration, and wound healing. However, high concentrations are significantly cytotoxic, impairing cellular proliferation, cellular migration, and wound healing in vitro.

## Background

Rotator cuff tendon tear, a cause of shoulder pain and dysfunction, is the most common shoulder disease that is closely related to the aging process [1–3]. Elderly patients, who are commonly candidates for rotator cuff repair to reduce their shoulder pain and improve their shoulder function, often additionally have osteoporosis of the proximal humerus [4, 5]. The suture anchor technique is currently popular for arthroscopic rotator cuff repair [6]. Several biomechanical studies have reported that inadequate bone mineral density (BMD) in the tuberosities of the proximal humerus negatively influences the critically important pullout strength of suture anchors, which determines the final fixation strength of inserted implants. BMD has been reported as an independent determining factor affecting postoperative rotator cuff healing [7]. Previous studies additionally suggest that reduced BMD is caused by excessive osteoclastic activity, which could impair bone ingrowth at the tendon-to-bone attachment sites [8, 9]. Therefore, the assessment of BMD and, if necessary, its improvement, and the suppression of osteoclastic activity are essential for rotator cuff repair in the elderly.

Bisphosphonates, despite their long-established use as the first line of treatment for osteoporosis, have concerning relationships to osteonecrosis of the jaw and atypical fracture of the femur [10–13]. Although bisphosphonates are known to suppress bone turnover, the effect of this action on soft tissues has not been completely evaluated. Some studies have reported that alendronate, a commonly used bisphosphonate, is cytotoxic to some cells, including oral keratinocytes [14], gingival fibroblasts [15], periodontal ligament fibroblasts [16], and endothelial cells [17]. However, no study has addressed the cytotoxicity of bisphosphonates on human rotator cuff tendon fibroblasts (HRFs). The purpose of this study was to evaluate the cytotoxicity of alendronate (Ald), a bisphosphonate, and its effects on HRF wound healing.

## Methods

### Study design

**Fig. 1:**
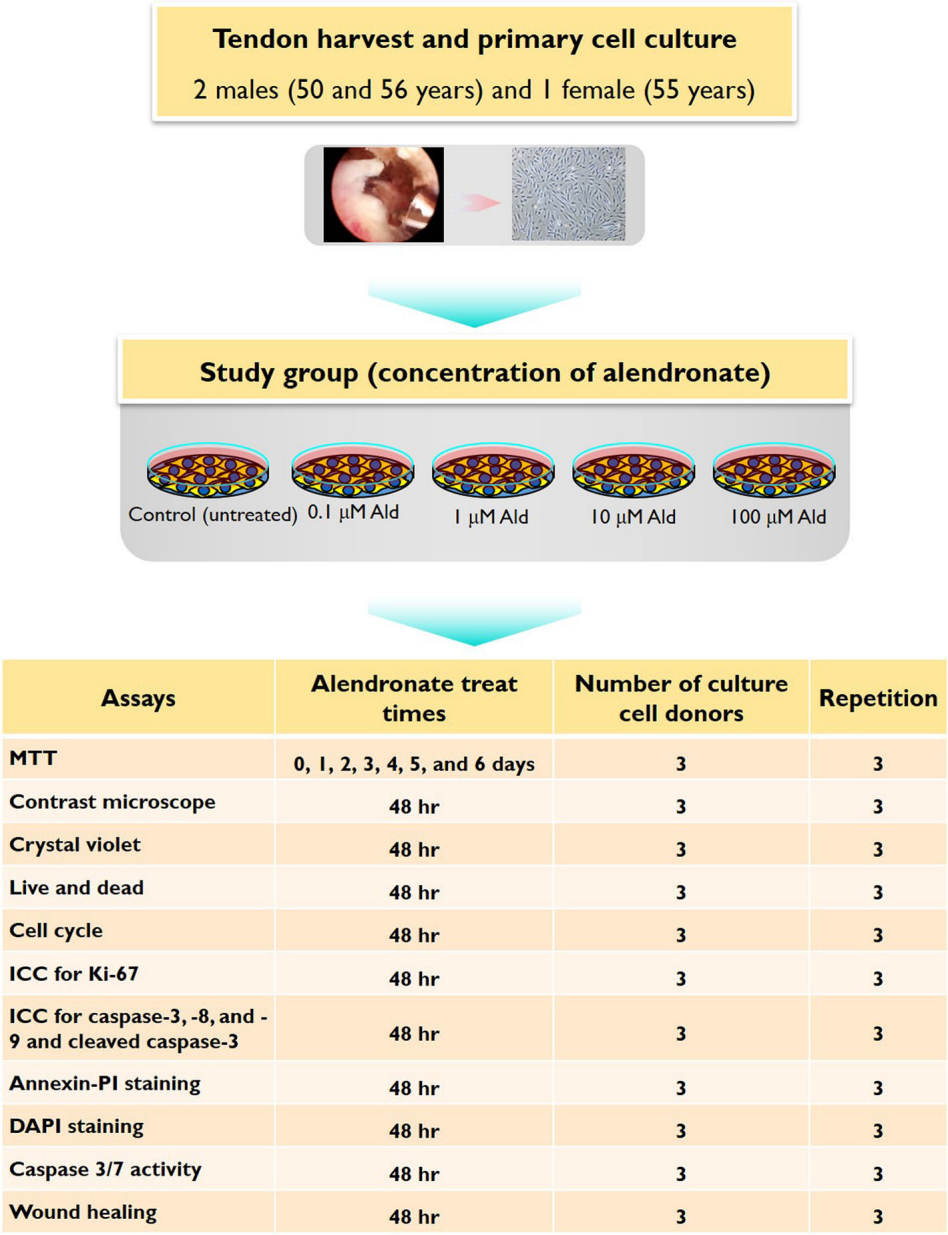
A schematic drawing of the experimental design, number of culture cell donors, and the repetition times of each test

This study used HRFs. This experiment used a control group and a study group composed of four subgroups, each treated with a different concentration of sodium alendronate (0.1, 1, 10, and 100 μM Ald) (Sigma, St. Louis, MO, USA). The concentrations were set by referring to several previously published studies which had used concentrations of alendronate [18–20]. Each study subgroup was exposed to its specific concentration of alendronate for 48 h, except during the measuring of the metabolism of 3-(4, 5-dimethyldiazol-2-yl)-2, 5-diphenyltetrazolium bromide (MTT), which was performed at varying times of 1 day to 6 days. These experimental groups were evaluated for cell viability, cell cycle and cell proliferation, type of cell death, caspase activity, and wound-healing ability. The schematic description of the study’s design, the number of samples, and the repetition times for each test are illustrated in Fig. 1.

### Cell culture

The human supraspinatus tendon tissues were collected from patients undergoing arthroscopic rotator cuff repair. The harvested tendon tissues were obtained from three patients (2 males and 1 female, ages 50, 56, and 55 years) from 2010 to 2011. The rotator cuff tears that were arthroscopically repaired were one small bursal-side partial-thickness tear and two full-thickness tears of medium and large sizes. Briefly, the tissues were washed twice with PBS (Lonza, Walkersville, MD, USA), minced into small pieces with a sterile scalpel, and placed on a 6-well tissue culture plate (Corning, Corning, NY, USA) in DMEM (Lonza) supplemented with 20% FBS (Gibco, Grand Island, NY, USA) and 1% Antibiotic-Antimycotic (Gibco) in a humidified 5% CO_2_ atmosphere at 37 °C. After two weeks, the cells had reached 90% confluence. The cells were then trypsinized (0.02% trypsin, 0.02% EDTA in PBS) for 5 min, centrifuged at 330 g for 3 min, and expanded in a second passage. The cells were then harvested with trypsin/EDTA and cryopreserved. For this experiment, third-passage cells were used.

### Cell viability analyses

Cell viability was estimated by measuring the MTT (Sigma). HRFs (2 × 10^4^) were seeded in each well of a 24-well plate. The cells were maintained in an incubator at 5% CO_2_, 37 °C for 24 h. The cells were treated with alendronate (0 μM, 0.1 μM, 1 μM, 10 μM, and 100 μM) for 1, 2, 3, 4, 5, and 6 days. A 500 μL MTT solution (0.5 mg/mL in free media) was briefly added to each well of the 24-well plate. Then, the plate was incubated for 2 h. Afterwards, the cell supernatant was removed and the solution of 200 μL DMSO (Merck, Darmstadt, Germany) was added to each well of the plate. Absorbance of the plate was measured at 570 nm, using a microplate reader. Cell viability was expressed as a percentage of live cells, compared with the control, which was set at 100%.

Cell viability analysis using the crystal violet assay was performed as follows. HRFs (1 × 10^5^) were seeded in each well of a 6-well plate. The cells were maintained in an incubator at 5% CO_2_, 37 °C for 24 h. After that, the cells were treated with alendronate (0 μM, 0.1 μM, 1 μM, 10 μM, and 100 μM) for 48 h. The cells were stained using 0.1% crystal violet solution (Sigma) for 2 h at room temperature, and then were washed with PBS. Then, the stained cells in the 6-well plate were analyzed using a scanner (PowerLook 2100XL, Umax, Dallas, TX, USA).

Cell viability was also assessed using the LIVE/DEAD Viability/Cytotoxicity Kit (Invitrogen, Carlsbad, CA, USA). HRFs (1 × 10^5^) were seeded in a 35 mm confocal dish. The cells were maintained in an incubator at 5% CO_2_, 37 °C for 24 h. The cells were treated with alendronate (0 μM, 0.1 μM, 1 μM, 10 μM, and 100 μM) for 48 h. Briefly, a Live/Dead kit solution (5X dye) was added to the dish. After the dish was incubated for 10 min at room temperature, the cells were evaluated using a laser-scanning confocal imaging system (IX70, Olympus, Tokyo, Japan); digital photographs were taken at 100 magnifications.

### Analyses for cell cycle and cell proliferation

Cell-cycle analyses using the PI (propidium iodide, Sigma) reagent were performed as follows. HRFs (1 × 10^5^) were seeded in each well of a 6-well plate. After 24 h of incubation, the experimental groups were exposed to alendronate (0 μM, 0.1 μM, 1 μM, 10 μM, and 100 μM) for 48 h. The cultured HRFs were harvested after trypsinization, and then collected after centrifugation. Those cells were washed with PBS, then fixed with 70% ethanol. They were then stained, using PBS containing 0.05 mg/mL PI and 1 μg/mL RNase, and 1 μg/mL Triton X-100. Flow cytometry (Cytomics FC500, Beckman Coulter, Fullerton, CA, USA) was used to measure the fluorescence intensity of each of the cells. Then, the subG1 population (cells with DNA fragmentation) was measured from the PI histogram.

Cell proliferation was evaluated with the Ki-67 staining method. HRFs (1 × 10^4^) were seeded on each well of a 24-well cell culture plate. Following treatment with alendronate, the cells were incubated for 48 h. The culture medium was removed from each well, and the cells were washed with PBS. A fixative solution of 4% paraformaldehyde was added to each well, which was then incubated for 20 min at 4 °C. The wells were then washed twice with PBS. The cells were permeabilized with 0.3% Triton X-100 added to each well, which was then incubated for 20 min at room temperature. The cells were then incubated in 5% bovine serum albumin (Amresco, Solon, OH, USA) in PBS for 1 h at room temperature. After that, the 1:300 diluted anti-Ki67 primary antibody (Ki-67 ab15580, Abcam, Cambridge, MA, USA) was added, and the cells were incubated for 2 h at room temperature. The wells were then washed twice with PBS. The secondary antibody (red) (goat anti-rabbit IgG, DyLight®550, A120-101D3, Bethyl, Montgomery, TX, USA) was used at the 1:500 dilutions for 1 h at room temperature and cells were counterstained with 1 μg/ml of DAPI (4′,6-diamidino-2-phenylindole, Sigma). The wells were washed again with PBS. The cells were then evaluated through a fluorescence microscope (ECLIPSE Ti-S, Nikon, Tokyo, Japan).

### Analyses for type of cell death

HRFs (1 × 10^5^) were seeded in each well of a 6-well plate. After 24 h of incubation, the experimental groups were exposed to alendronate (0, 0.1, 1, 10, and 100 μM) for 48 h. The cultured HRFs were harvested after trypsinization, and then collected after centrifugation. Those cells were washed with PBS, then stained using a fluorescein isothiocyanate (FITC) Annexin V-PI kit (BD Biosciences, San Diego, CA, USA), according to the manufacturer’s instructions. Using flow cytometry (Cytomics FC500, Beckman Coulter), cell viability was determined as follows: live cells were labeled with neither stain; apoptotic cells were labeled only with Annexin V; and necrotic cells were labeled with both Annexin V and PI.

DAPI (4′, 6-diamidino-2-phenylindole) staining for evaluation of DNA fragmentation was performed as follows. HRFs (1 × 10^5^) were seeded in each well of a 6-well plate. After 24 h of incubation, the experimental groups were exposed to alendronate (0, 0.1, 1, 10, and 100 μM) for 48 h. After washing with PBS, the cells were fixed with methanol for 5 min at − 20 °C and were then washed with cold PBS. The cells were kept in 1% triton X-100 in PBS solution for 10 min at room temperature and were then washed with PBS. The cells were stained with 1 μg/mL DAPI staining solution (Sigma) for 5 min at 37 °C and were then washed with PBS. The cells were evaluated using a fluorescence microscope (Nikon).

### Analyses of Caspases activity

HRFs (3 × 10^3^) were seeded in each well of a 96-well cell culture white plate; then the HRFs were incubated for 24 h. Then, after treatment with alendronate (0, 0.1, 1, 10, and 100 μM), the cells were incubated for 48 h. Then, caspase-3/7 activity was measured, using the Caspase-Glo® 3/7 Assay Kit, according to the manufacturer’s guide (Promega, Madison, WI, USA).

Cleaved caspase-3 activity was also evaluated morphologically, using immunocytochemistry. HRFs (1 × 10^4^) were seeded on the cover glass of each well of a 24-well cell culture plate. Then, after treatment with alendronate (0, 0.1, 1, 10, and 100 μM), the cells were incubated for 48 h. The culture media was removed from each well, and the cells were washed with PBS. A fixative solution of 4% paraformaldehyde was added to each well, and then washed twice with PBS. A 0.3% Triton X-100 in PBS solution was added to each well, which was then incubated for 20 min at room temperature. The wells were then washed twice with PBS, and the cells were incubated in 5% bovine serum albumin (Amresco) in PBS for 1 h at room temperature. Then, the 1:200 diluted primary antibodies against cleaved caspase-3 (Asp175, Cell Signaling Technology, Beverly, MA, USA) were added, and the cells were incubated overnight at 4 °C. The cells were then washed twice with PBS. The cells were stained with a goat anti-rabbit IgG secondary antibody (A120-101D3, Bethyl) and counterstained with DAPI. The cells were then evaluated, using a fluorescence microscope (Nikon) under 200 magnifications. To identify the apoptosis pathways, we evaluated the activities of caspase-8 representing the extrinsic pathway and the activities of caspase-9 representing the intrinsic pathway, using the same methodological sequence for each primary antibody of caspase-8 (ab25901, Abcam) and caspase-9 (ab47537, Abcam).

### Wound-healing analyses

Wound-healing was examined with a scratch assay, using a Culture-Insert (ibidi, Munich, Germany). HRFs (7 × 10^4^) were seeded in each Culture-Insert. The cells were maintained in an incubator at 5% CO_2_ and 37 °C for 24 h. The study groups were treated with alendronate (0, 0.1, 1, 10, and 100 μM) for 48 h prior to wounding. After removal of the Culture-Insert, the wounds were created according to a standardized protocol, using a trimmed comb with a cell-free gap of 500 μm. Immediately after wounding, a brief wash was performed, and the culture media was added to the entire experimental group. With a phase contrast microscope, migration of cells into wounded areas of the plate was observed at 0 h, 12 h, 24 h, and 48 h. Nine fields of the wounded area were photographed at 40 magnifications. For each experimental group, the areas not covered by cells at the exposure times of 0 h, 12 h, 24 h, and 48 h were determined by analysis with the Stream image (Olympus). The migration area’s percentage in each subgroup was calculated by determining the difference between the 0-h and 48-h areas, and then dividing that by the difference in the control during the two time points, × 100.

### Statistical analyses

Each experiment was performed at least three times; the results were presented as the mean of the total number of trials performed, in order to obtain more objective data. All values were expressed as mean ± standard deviation (SD). All statistical analyses were performed via one-way ANOVA, followed by Tukey’s post hoc test. Differences with a probability of less than 0.05 were considered statistically significant. All statistical analyses were done by SPSS 20.0 for Windows (SPSS, Chicago, IL, USA).

## Results

### Cytotoxicity analyses

**Fig. 2:**
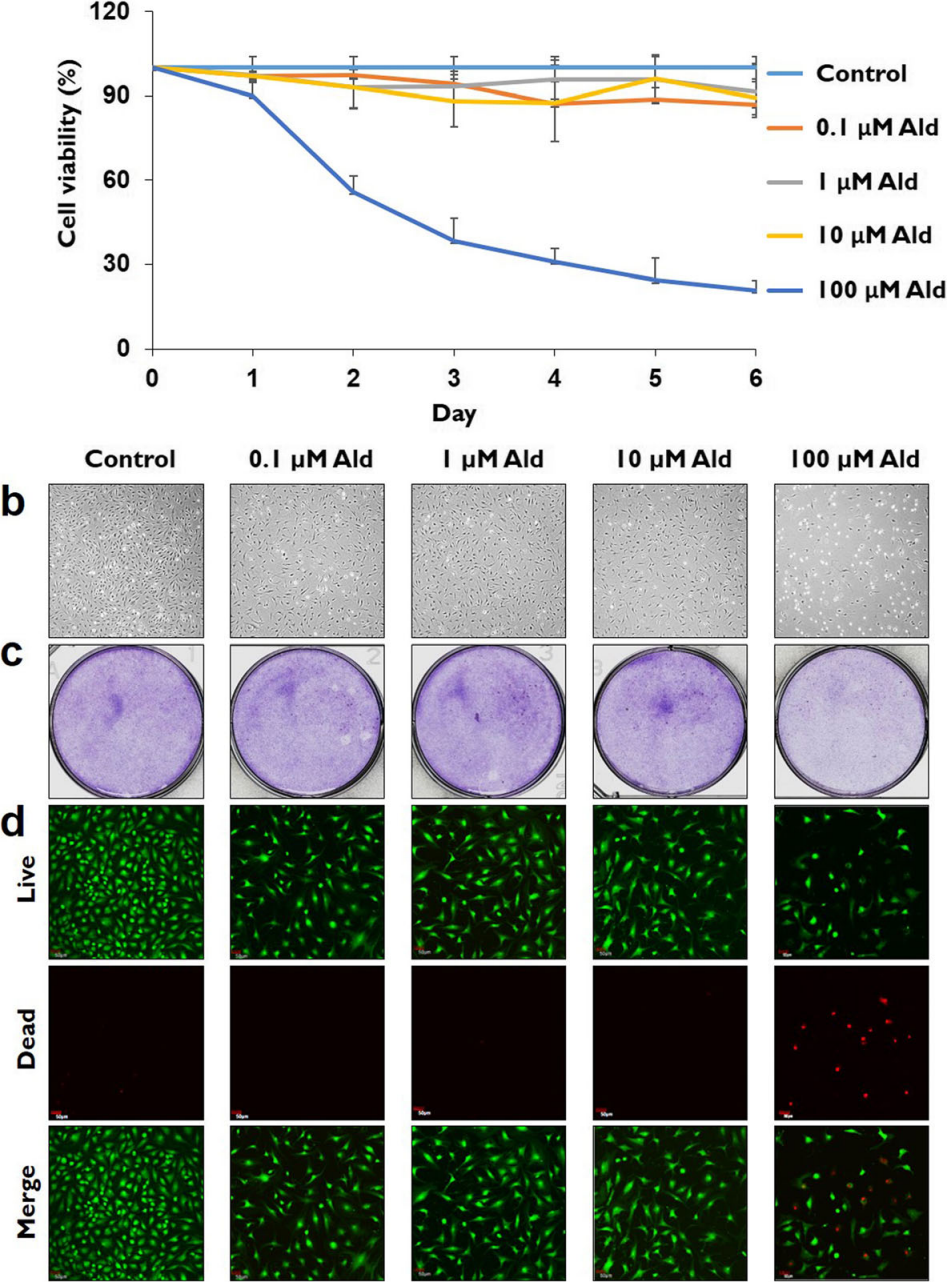
Cell viability analyses. **a** Cell viability was significantly decreased in a time-dependent manner in 100 μM Ald group, the group with the highest alendronate concentration (*p* < 0.001). After 24 h, cell viability in 100 μM Ald was significantly lower than in the other studied groups (*P* < 0.001). **b** Contrast microscope analyses demonstrated that the cell population in 100 μM Ald group was markedly more decreased than in the other studied groups. **c** Crystal violet stain analyses confirmed that live cells were markedly more decreased in 100 μM Ald group than in the other studied groups. **d** The live and dead assays showed that the population of live cells (green) was more decreased and the population of dead cells (red) was more increased in 100 μM Ald group than in the other studied groups

Cultures treated with the highest alendronate concentration (100 μM Ald) showed significantly lower percentages of live cells than the other groups (control, 0.1, 1 and 10 μM Ald) after 24 h (*p* < 0.001). The rate of live cells in the 100 μM Ald group significantly decreased with time dependence (*p* < 0.001), reaching 24.53 ± 7.71% (mean ± SD) of total cells in 5 days. The rates of live cells in control, 0.1, 1 and 10 μM Ald did not differ significantly from one another (*p* > 0.05) (Fig. 2a).

According to the morphological analyses using phase contrast microscope and crystal violet staining, the cell population was markedly more decreased in the 100 μM Ald group than in the other studied groups (Fig. 2b and c). According to the cell viability analyses using a LIVE/DEAD kit, the number of dead cells stained with red color in the 100 μM Ald group was markedly higher than in the control group and the other studied groups (0.1, 1 and 10 μM Ald) (Fig. 2d).

### Analyses of cell cycle and cellular proliferation

**Fig. 3:**
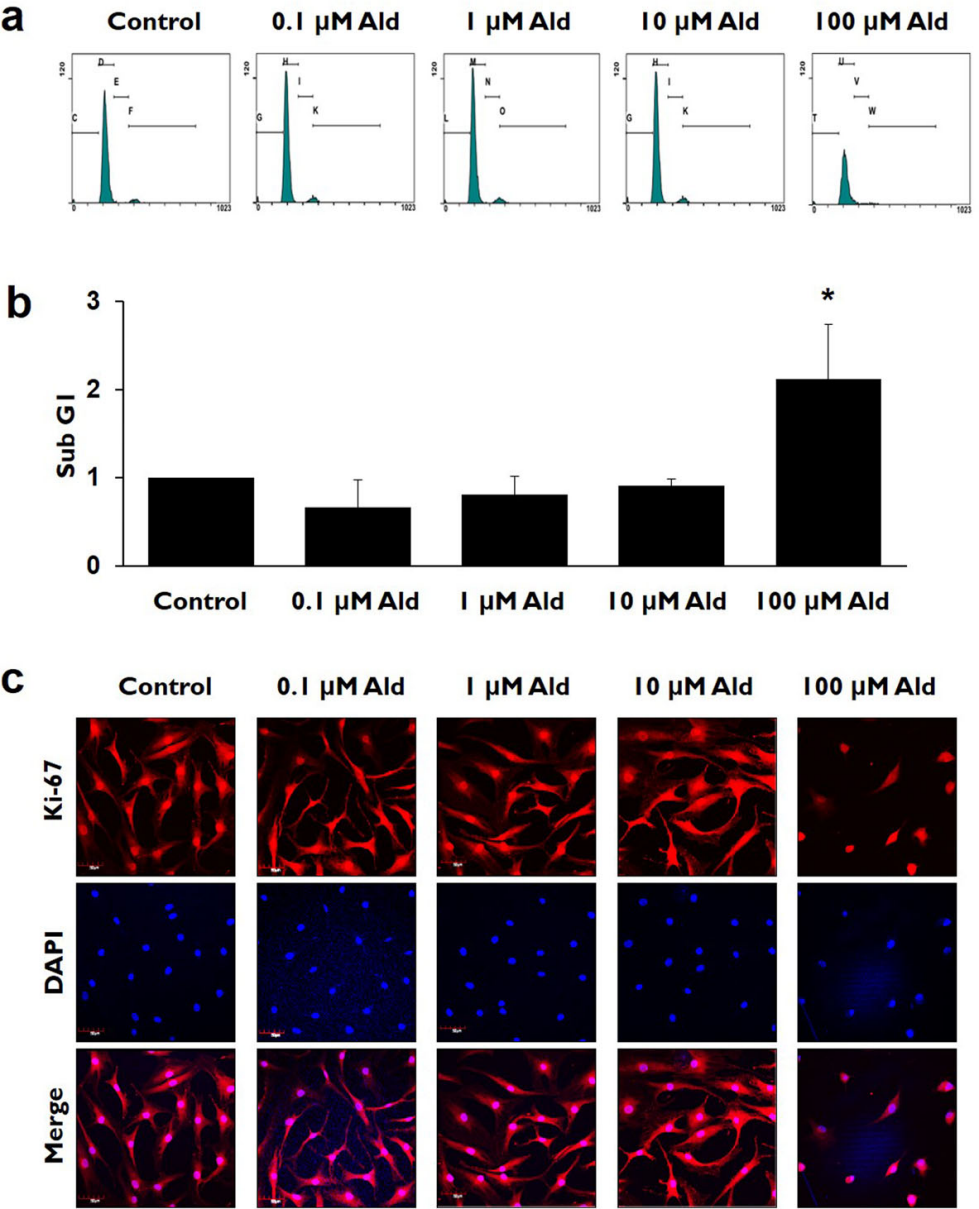
The comparisons of cell cycle and proliferation rates in various study groups. **a** and **b** The cell population of subG1 increased significantly in 100 μM Ald group, as compared with the control and the other studied groups (*p* ≤ 0.013). * represents the *p* values that are less than 0.05. **c** Immunocytochemical staining demonstrates that the Ki-67 positive cells representing cellular proliferation markedly decreased in 100 μM Ald group, as compared with the control and the other studied groups

The subG1 population increased significantly in the 100 μM Ald group, as compared with the control and the other studied groups, according to the FACS analyses using PI staining (*p* ≤ 0.013). The subG1 populations in the control, 0.1, 1, and 10 μM Ald groups did not differ significantly from each other (*p* > 0.05) (Fig. 3a and b).

Cellular proliferation shown by Ki-67 positive cells was more decreased in the 100 μM Ald group than in the control and the other studied groups (Fig. 3c).

### Analysis of Annexin V-PI and DAPI staining

**Fig. 4:**
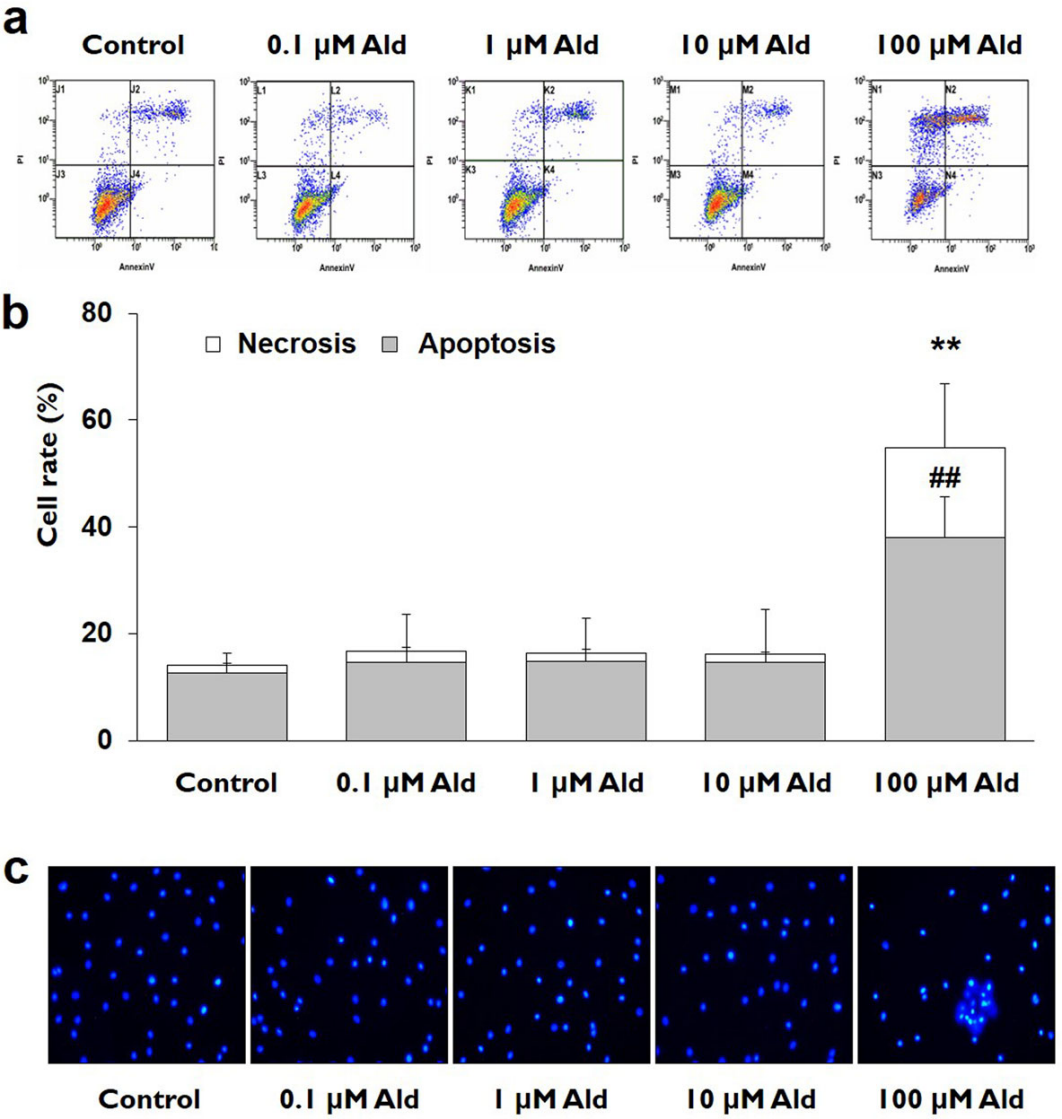
Analyses for type of cell death in various study groups. **a** and **b** In the Annexin V-PI double staining analyses, the rates of apoptosis and necrosis in 100 μM Ald group were significantly higher than in the control and the other studied groups (*p* < 0.01). ## signifies the *p* values of the apoptosis that are less than 0.01 and ** represents the *p* vlaues of the necrosis that are less than 0.01. **c** In the DAPI staining analyses, DNA fragmentation, a marker for apoptosis, was markedly increased in 100 μM Ald group as compared with control and the other studied groups

The mean percentages of apoptotic and necrotic cells were significantly higher in the 100 μM Ald group than in the control and the other studied groups, according to the FACS analyses using Annexin V-PI double staining (*p* < 0.01). The mean percentages of apoptotic and necrotic cells did not differ significantly among the control and the other studied groups (*p* > 0.05) (Fig. 4a and b).

The number of cells with DNA fragmentation was higher in the 100 μM Ald group than in the control and the other studied groups, according to the DAPI staining analyses. The numbers of cells with DNA fragmentation did not differ markedly among control and the other studied groups (Fig. 4c).

### Caspases activity assay

**Fig. 5:**
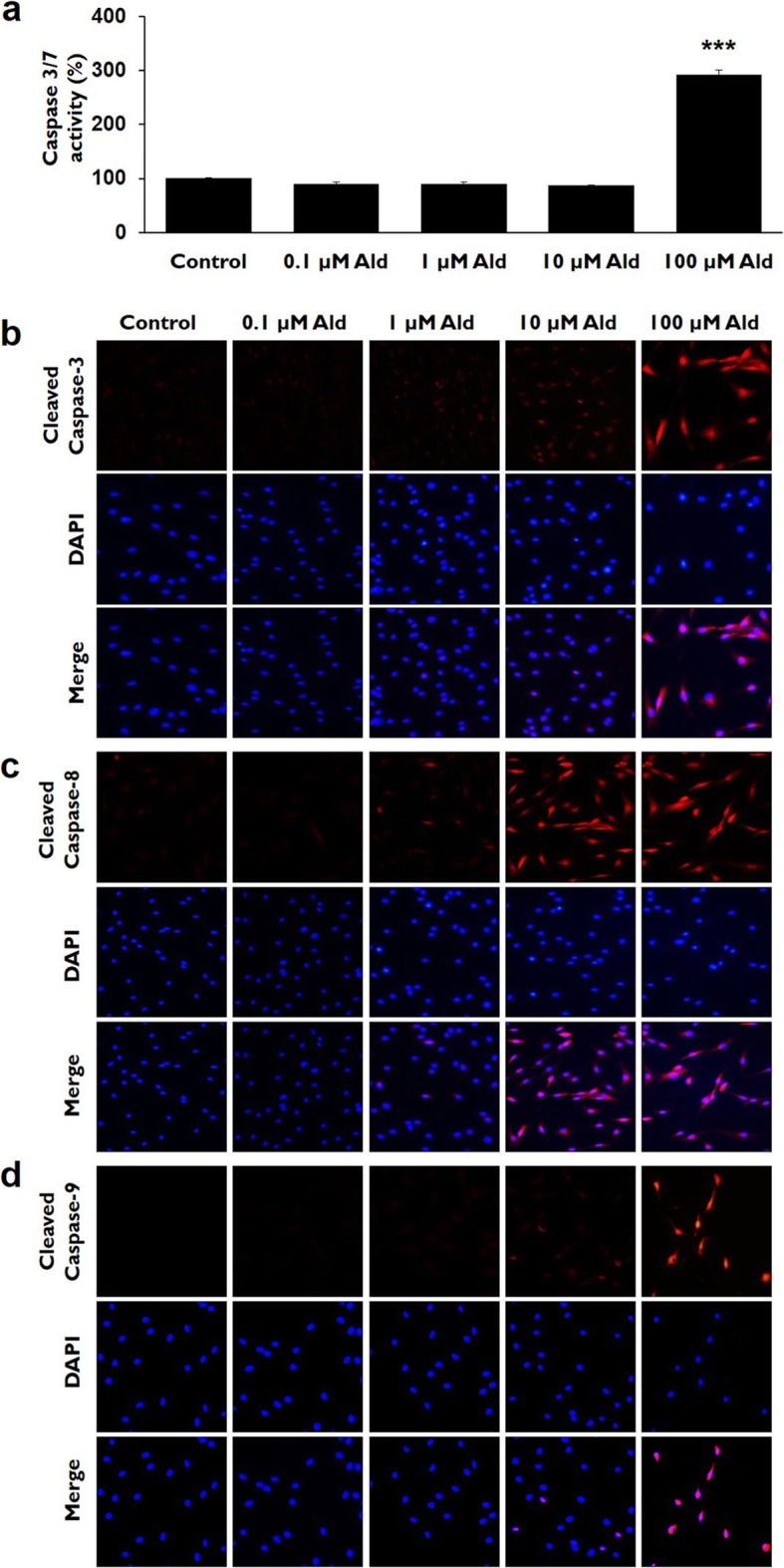
Caspase-3/7 activity and immunocytochemistry for caspase-3, caspase-8, and caspase-9 activity. **a** Caspase-3/7 activity was significantly higher in 100 μM Ald group, as compared with control and the other studied groups (*p* < 0.001). *** represents the p vlaues that are less than 0.001. **b** Cleaved caspase-3 expression (red) was higher in 100 μM Ald group than in control and the other studied groups in the immunocytochemistry study. **c** Caspase-8 expression (red) in 100 μM Ald group was higher than in control and the other studied groups. **d** Caspase-9 expression (red) in 100 μM Ald group was higher than in control and the other studied groups

Caspase-3/7 activity in the 100 μM Ald group was significantly higher than in control and the other studied groups (*p* <0.001). Caspase-3/7 activity did not differ significantly among the control and the other studied groups (*p* > 0.05) (Fig. 5a). The comparatively higher caspase-3 expression in the 100 μM Ald group was confirmed by immunocytochemistry staining (Fig. 5b); Caspase-8 expression in the 100 μM Ald group was higher than in control and the other studied groups (Fig. 5c); Caspase-9 expression in the 100 μM Ald group was also higher than in control and the other studied groups (Fig. 5d).

### Wound-healing analyses

**Fig. 6:**
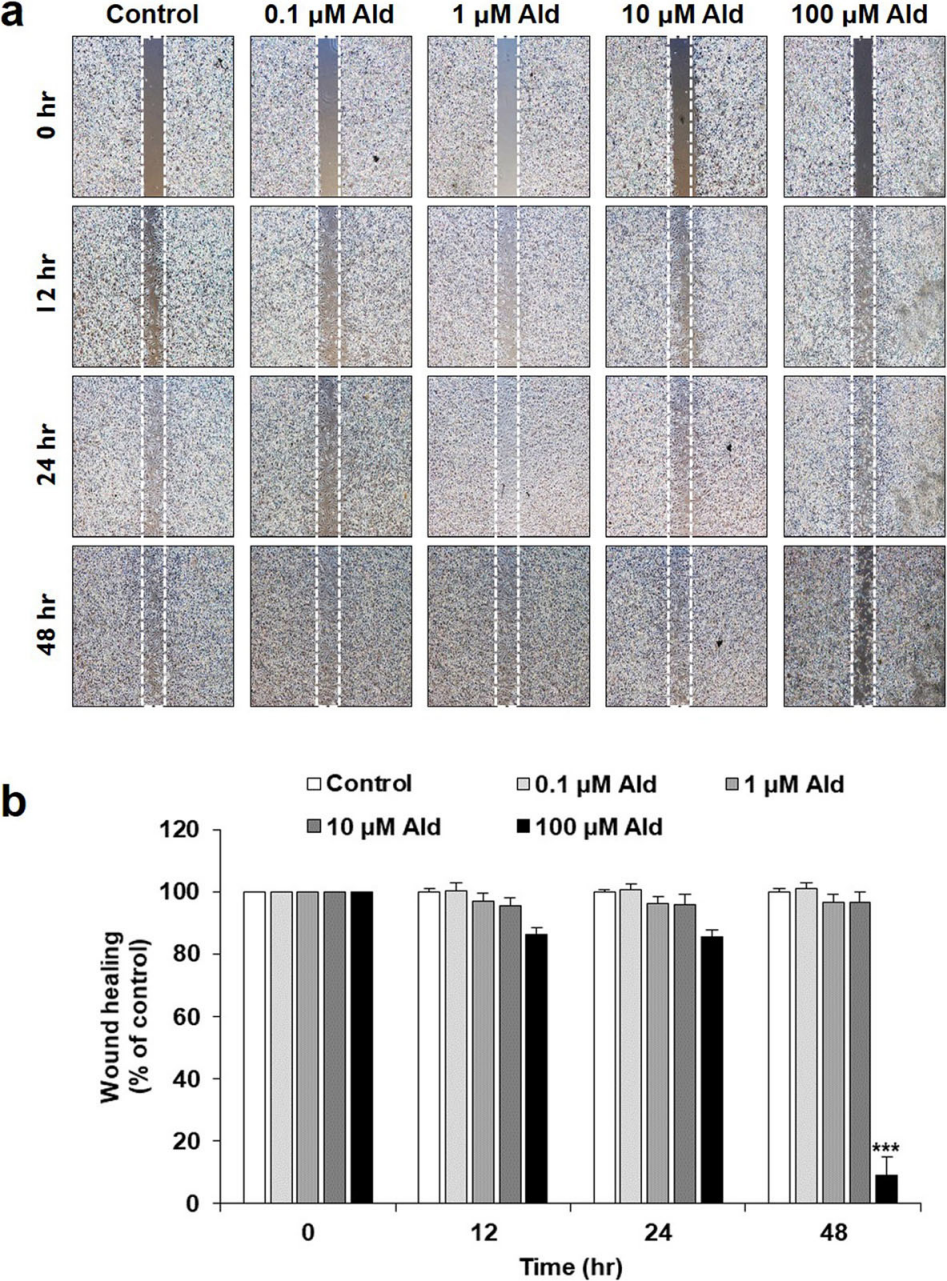
Wound-healing analyses. **a** In a comparison of scratch wound-healing, all the wounds healed within 48 h, except in 100 μM Ald. **b** The percentage of wound healing in 100 μM Ald group was significantly lower than in control and the other studied groups (*p* < 0.001). *** represents the p vlaues that are less than 0.001. The mean percentages of wound-healing in control, 0.1, 1 and 10 μM Ald group did not differ significantly from one another (*p* > 0.05)

In a comparison of scratch-wound healing in a 5% FBS media, all the wounds healed within 48 h, except in the 100 μM Ald group. The mean values in each group were control, 100.00 ± 1.25; 0.1 μM Ald, 101.01 ± 1.99; 1 μM Ald, 96.84 ± 2.39; 10 μM Ald, 96.56 ± 3.45; and 100 μM Ald, 9.04 ± 5.75. The wound-healing results in the 100 μM Ald group were significantly lower than in the other studied groups and the control (*p* < 0.001). The mean percentages of wound healing in the control, 0.1, 1 and 10 μM Ald groups were not significantly different from each other (*p* > 0.05) (Fig. 6a and b).

## Discussion

The notable finding of the current study was that a high concentration of alendronate (100 μM Ald) has a cytotoxic effect on HRF. In this study, cell viability was significantly decreased in the subgroup with the highest alendronate concentration, as compared to the control and the other studied subgroups. The decreased cell viability was mainly caused by apoptosis involving the caspase-3 pathway. Alendronate-induced cytotoxicity has been reported in several studies involving oral keratinocytes [18], gingival fibroblasts [21], human periodontal ligament fibroblasts [18], endothelial cells [22], and human bone-marrow-derived stem cells [23]. Those studies all found that the highest concentrations of alendronate, ranging from 10^−11^ M to 100 M, had various levels of cytotoxicity to the studied cells. Those findings support the results of this study.

This study showed that the population of subG1 cells increased significantly in the 100 μM Ald group, as compared with the control and other studied groups. This means that many cells did not actively participate in cellular proliferation due to DNA fragmentation. We also found that 100 μM Ald inhibited cellular proliferation, using immunocytochemistry for the Ki-67 antigen, which is a well-known marker for cell proliferation. The Ki-67 antigen is always present in proliferating cells in all active phases of the cell cycle (G_1_, S, G_2_, and mitosis), but is absent from resting cells (G_0_) [24]. All these findings suggest that the highest concentration of alendronate inhibited cellular proliferation.

Although previous studies reported that, in high concentrations and in a time-dependent manner, alendronate has cytotoxic effects, those studies did not evaluate the types or the molecular mechanisms of alendronate-induced cell death. We evaluated the types of alendronate-induced cell death and determined that apoptosis is the predominant type. Although we did not evaluate in detail the cascade mechanisms of apoptosis related to alendronate, we determined that caspase-3, a well-known executioner of cells in the apoptosis mechanism, operates in alendronate-induced cytotoxicity to HRFs [25, 26]. We also evaluated caspase-8, involving extrinsic apoptosis pathways, and caspase-9, involving intrinsic pathways [27, 28]. In this study, both caspases were highly expressed in the group with the highest concentration (100 μM Ald). That means that both extrinsic and intrinsic apoptosis pathways are involved in alendronate-induced apoptosis.

The wound-healing analysis indicated that the highest concentration of alendronate reduced wound-healing, not only affecting cell survival but also inhibiting cell proliferation and cell migration, which are processes vital to rotator cuff healing. One study that evaluated alendronate’s effect on wound-healing used a rat gastric epithelial cell line [29]. That study reported that alendronate did not affect the wound-healing ability of rat gastric epithelial cells; however, in a coculture with endothelial-derived growth factor and alendronate, the wound-healing ability was less than with endothelial-derived growth factor alone. That study indicated that alendronate diminishes the endothelial-derived growth factor’s wound-healing effect [29]. The current and previous studies suggest that, above a certain concentration, alendronate has a negative effect on wound healing. In animal experiments, the effects of bisphosphonates for tendon-to-bone healing are controversial. Thomopoulos et al. [30] reported that alendronate prevents bone loss and improves tendon-to-bone repair. However, not only did alendronate not improve tendon stiffness, but systemic plus local treatment of alendronate was detrimental to stiffness of the repair due to alendronate’s effect on the inflammatory process. Thomopoulos et al. [30] suggested that the high local dose of alendronate may have led to an enhanced inflammatory reaction in the bone tunnel that was detrimental to the healing process. Lui et al. [31] reported that alendronate reduced peri-tunnel bone resorption, increased mineralized tissue inside the bone tunnel, and histologically and biomechanically promoted graft-bone tunnel healing. That study also reported that alendronate might be used for reducing peri-tunnel bone loss and promoting graft-bone tunnel healing at the early stage of anterior cruciate ligament (ACL) reconstruction. The Lui et al. [31] study’s result differs from those of the Thomopoulos et al. [30] study. Conversely, Hjorthaug et al. [32] showed a negative effect of a bisphosphonate, in which zoledronic acid reduced pullout strength and the stiffness of the tendon-bone interface. That study reported no evidence supporting the use of zoledronic acid as adjuvant treatment in tendon-to-bone healing. There is still controversy as to whether bisphosphonates enhance tendon-to-bone healing. However, those previous studies may not be directly comparable to our study due to the differences in the study designs.

This study has several limitations. We did not evaluate every bisphosphonate that is used in clinical practice because most injectable bisphosphonates are used to treat metastatic cancer and are not practical for patients needing rotator cuff repair [33]. We focused on alendronate, a lower-potency bisphosphonate commonly used to treat osteoporosis, giving this study clinical significance for rotator cuff surgery [34, 35]. Although this study demonstrated the cytotoxicity of alendronate in a high concentration, the authors did not determine whether the studied high concentration (100 μM Ald) of alendronate is within the range of physiological concentrations of rotator cuff tendon tissue used in clinical practice. Furthermore, we did not determine alendronate’s distribution in the different types of tissue, especially rotator cuff tendon tissue. Therefore, the appropriateness of immediate alendronate treatment for patients following rotator cuff tendon repair should be further evaluated in animal-model or clinical research.

## Conclusion

Low concentrations of alendronate appear to have little effect on HRF viability, proliferation, migration, and wound healing. However, high concentrations are significantly cytotoxic, impairing cellular proliferation, cellular migration, and wound healing in vitro.

## Data Availability

The dataset generated and/or analyzed during the current study is not publicly available due to legal and ethical considerations. However, the data are available from the corresponding author, upon reasonable request.

## Abbreviations

Ald: Alendronate
BMD: Bone mineral density
FITC: Fluorescein isothiocyanate
HRFs: Human rotator cuff tendon fibroblasts
MTT: Metabolism of 3-(4, 5-dimethyldiazol-2-yl)-2, 5-diphenyltetrazolium bromide
SD: Standard deviation
